# An unusual case of central diabetes insipidus & hyperglycemic hyperosmolar state following cardiorespiratory arrest

**DOI:** 10.1186/1756-0500-6-325

**Published:** 2013-08-16

**Authors:** Muhammad Qamar Masood, Suneel Kumar, Ali Asghar, Abdul Jabbar

**Affiliations:** 1 Department of Medicine, Section of Endocrinology, Aga Khan University Hospital, Stadium Road, P.O. Box 3500, Karachi 74800, Pakistan; 2 Department of Medicine, Endocrinology, Sultan Qaboos University Hospital, Muscat, Oman

**Keywords:** Central, Diabetes, Insipidus, Hyperosmolar, Hyperglycemic, State, Cardiopulmonary, Arrest

## Abstract

**Background:**

We are describing an unusual case of severe hyperglycemia and hypernatremia, resistant to treatment.

**Case presentation:**

A thirty year old female with adenocarcinoma of rectum was admitted with increasing lethargy, headache and drowsiness. She deteriorated rapidly and had cardiac arrest, following which she remained comatose. Her initial serum glucose and sodium were normal, but after receiving dexamethasone and mannitol, the serum glucose progressively increased to 54.7 mmol/L and sodium to 175 mmol/L, despite receiving very high dose of intravenous (IV) insulin infusion. She was evaluated for diabetes insipidus because of continued polyuria even after correction of hyperglycemia. Her serum osmolality was 337 mmol/kg, and urine osmolality was 141 mmol/kg which rose to 382 mmol/kg, after receiving 4 mcg of IV Desmopressin.

**Conclusion:**

Our patient developed central diabetes insipidus post cardiac arrest and severe dehydration because of diabetes insipidus. Stress of critical illness, dehydration, dexamethasone and IV dextrose infusion were likely responsible for this degree of severe and resistant to treatment hyperglycemia.

## Background

Central diabetes insipidus (DI) is characterized by decreased release of antidiuretic hormone (ADH), resulting in a variable degree of polyuria. Lack of ADH can be caused by neurosurgery or trauma, primary or secondary tumors, infiltrative diseases, idiopathic DI, hypoxic encephalopathy or severe ischemia. Cases of DI are described in literature in association with cardiopulmonary arrest, severe hypoxia secondary to drug induced respiratory failure as well as carbon monoxide poisoning [1-3].

## Case presentation

Our patient was a 30 year old female recently diagnosed with adenocarcinoma of rectum with metastasis to inguinal lymph nodes. She underwent diversion colostomy. The procedure and hospital course was uneventful, and she was discharged two days later with further plan for chemotherapy and radiation therapy two weeks post surgery.

Twelve days later, she presented in emergency department at 2230 hours with complains of headache and progressive lethargy. She was drowsy but arousable; otherwise her neurological and systemic examination was unremarkable. Laboratory data showed leucocytosis with left shift, mild hypercalcemia and hypokalemia. Serum sodium and glucose were normal (Table 1). She was given IV fluids with normal saline, potassium replacement and Pamidronate infusion. IV hydrocortisone 200 mg, Ceftriaxone and acyclovir were given emperically. MRI Brain and CSF analysis were normal.

**Table 1:** Laboratory parameters

**Test**	**Emergency room**	**Day 1**	**Day 2**		**Day 3**	
			**0600 hours**	**1500 hours**	**After IV fluids**	**After desmospressin**
Glucose (mmol/L)	6.7	“Hi” on CBG	“Hi” on CBG	54.7	9.2	
Sodium (mmol/L)	146	165	170	175	164	140
Potassium (mmol/L)	2.0	3.9	2.4	2.8	4.7	5.3
Chloride (mmol/L)	108	130	145		143	
Bircarbonate (mmol/L)	23.8	20.7	19.7		17.7	
Serum Osmolality (mmol/Kg)					337	294
Urine Osmolality (mmol/Kg)					141	382
Spot Urine Na (mmol/L)					52	136
BUN (mmol/L)	7.8	9.6	10.7		2.5	
Creatinine (μmol/L)	70.7	88.4	97.2		44.2	
Calcium (mmol/L)	2.75	2.92			2.20	
Total Intake (Liters per 24 hours)		0.6 (Only 3 hours)	8.7		18.8	
Urine output (Liters per 24 hours)		1.5 (Only 3 hours)	6.7		7.2	

Next day, she deteriorated rapidly with increasing restlessness, incoherent speech, followed by tonic clonic seizure. Later in the evening, she had cardiac arrest for which cardiopulmonary resuscitation (CPR) was done for three minutes.

She remained comatose after this episode. She was loaded with Phenytoin and started on IV dexamethasone and mannitol. She was also given isotonic saline boluses, plasma expanders (gelatin polypeptide), and dopamine and norepinephrine infusion.

On the same day, her capillary blood glucose (CBG) was noted to be more than 16 mmol/L. She was started on IV insulin infusion at 10 units per hour via insulin syringe pump, which was progressively increased but CBG remained HI (>27 mmol/L) on glucometer. After 24 hours on IV insulin infusion, her serum glucose (SG) was 54.7 mmol/L while she was still on 60 units per hour IV insulin infusion. Her serum sodium (Na) after cardiac arrest rose to 165 mmol/L. She was given IV Dextrose water 5% (D5W). Despite that, next day Na reached to 170 mmol/L while SG was 54.7 mmol/L (see Table 1). She was making urine about 250–400 mL/hour while receiving similar amount of IV fluids.

An assessment of hyperosmolar hyperglycemic state with severe dehydration was made. Glucocorticoids and stress of acute illness were thought to be responsible for initial rise in glucose and later osmotic diuresis because of mannitol and glycosuria resulted in severe dehydration and hypernatremia.

Patient was treated with IV 0.45% saline and D5W (total of eight liters overnight). After receiving fluids, her CBG gradually decreased to 9.2 mmol/L and insulin dose was titrated down to 18 units per hour. Her Na also decreased to 164 mmol/L. However after achieving near normal glycemia and alleviating osmotic diuresis, her urine output remained elevated (approximately 400 mL/hour). She was then evaluated for diabetes insipidus.

Her serum osmolality was 337 mmol/kg and urine osmolality was 141 mmol/kg. Urine osmolality rose to 382 mmol/kg after receiving 4 mcg of IV desmopressin. After desmopressin, her urine output dropped and serum Na gradually improved to 140 mmol/L. After desmopressin, her insulin requirement substantially decreased and she was taken off IV insulin. After correction of her metabolic derangements, she remained comatose and totally dependent on ventilator. At that point, the family decided to withdraw the support.

## Discussion

We believe that hypoxic damage to the posterior pituitary and/or hypothalamus during cardiac arrest was responsible for the central DI in this patient. A urine osmolality of 141 mmol/kg when serum osmolality was 337 mmol/kg was diagnostic of DI and response to desmopressin confirmed the central cause.

DI following cardiorespiratory arrest is a rare event and has been described in post cardiopulmonary arrest patients [1-4]. In the cases described by Udoshi et al. [4], no lesion was found in the posterior pituitary and hypothalamus on autopsy, however the second patient had infarction of anterior pituitary. Hypothalamic dysfunction i.e. wide fluctuations in heart rate, blood pressure and body temperature were noted in both patients.

DI has also been reported with pituitary tumor apoplexy [5], after moderate to severe traumatic brain injury [6], acute sheehan syndrome [7] and in patients with long standing hypopituitarism secondary to sheehan syndrome [8].

A case of non-ketotic hyperglycemic coma in an 18 year old male, with previously undiagnosed diabetes mellitus, was described in association with hemorrhagic pituitary apoplexy. MRI in this patient revealed a heterogeneous mass, a prolactinoma, arising from an expanded sella turcica and extending into the suprasellar cistern [9]. In the two cases described by Udoshi et al. [4], both developed profound hyperglycemia.

Stress and drug induced hyperglycemia was culminated in profound and resistant to treatment hyperglycemia in our patient. This was due to extreme dehydration secondary to DI, and the hyperglycemia mainly responded to fluid resuscitation.

## Conclusion

Our patient developed central DI post cardiac arrest manifested as hypernatremia and severe dehydration, responsible for this degree of severe and resistant to treatment hyperglycemia.

## Consent

Written informed consent was obtained from the patient’s brother (next of kin) for publication of this case report. A copy of the written consent is available for review by the Series Editor of this journal.

## Abbreviations

IV: Intravenous; DI: Diabetes insipidus; ADH: Antidiuretic hormone; CPR: Cardiopulmonary resuscitation; CBG: Capillary blood glucose; SG: Serum glucose; Na: Serum sodium; D5W: Dextrose water 5%.

## Competing interests

The authors declare that they have no competing interests.

## Authors’ contributions

QM gave the concept of research paper, and critically reviewed the manuscript. SK led the acquisition of data, review of literature, and drafted the manuscript. AA reviewed the manuscript. AJ critically reviewed the manuscript. All authors read and approved the manuscript.

## Authors’ information

QM is fellow of the American College of Physicians & the American College of Endocrinology. He is Assistant Professor in Diabetes, Endocrinology & Metabolism, Department of Medicine, Aga Khan University Hospital. He was involved in the medical management of the patient. SK is fellow of the College of Physicians & Surgeons of Pakistan. He is Senior Instructor in Endocrinology, Department of Medicine, Sultan Qaboos University Hospital. He was involved in the medical management of the patient. AA is member of the Royal Colleges of Physicians of the United Kingdom. He is Fellow in Endocrinology, Diabetes & Metabolism, Department of Medicine, Aga Khan University Hospital. He was involved in the medical management of the patient. AJ is fellow of the Royal College of Physicians of London. He is Professor in Diabetes, Endocrinology & Metabolism, Department of Medicine, Aga Khan University Hospital.
